# Gender Differences in the Relationship between Maladaptive Behaviors and Post-Traumatic Stress Disorder. A Study on 900 L’ Aquila 2009 Earthquake Survivors

**DOI:** 10.3389/fpsyt.2012.00111

**Published:** 2013-01-04

**Authors:** Liliana Dell’Osso, Claudia Carmassi, Paolo Stratta, Gabriele Massimetti, Kareen K. Akiskal, Hagop S. Akiskal, Icro Maremmani, Alessandro Rossi

**Affiliations:** ^1^Section of Psychiatry, Department of Clinical and Experimental Medicine, University of PisaPisa, Italy; ^2^Section of Psychiatry, Department of Experimental Medicine, University of L’AquilaL’Aquila, Italy; ^3^International Mood Center, University of California at San DiegoLa Jolla, CA, USA

**Keywords:** PTSD, post-traumatic stress symptoms, earthquake, maladaptive behaviors, gender

## Abstract

**Background**: Post-traumatic stress disorder (PTSD) represents one of the most frequently psychiatric sequelae to earthquake exposure. Increasing evidence suggests the onset of maladaptive behaviors among veterans and adolescents with PTSD, with specific gender differences emerging in the latter. Aims of the present study were to investigate the relationships between maladaptive behaviors and PTSD in earthquake survivors, besides the gender differences in the type and prevalence of maladaptive behaviors and their association with PTSD. **Methods**: 900 residents of the town of L’Aquila who experienced the earthquake of April 6th 2009 (Richter Magnitude 6.3) were assessed by means of the Trauma and Loss Spectrum-Self Report (TALS-SR). **Results**: Significantly higher maladaptive behavior prevalence rates were found among subjects with PTSD. A statistically significant association was found between male gender and the presence of at least one maladaptive behavior among PTSD survivors. Further, among survivors with PTSD significant correlations emerged between maladaptive coping and symptoms of re-experiencing, avoidance and numbing, and arousal in women, while only between maladaptive coping and avoidance and numbing in men. **Conclusions**: Our results show high rates of maladaptive behaviors among earthquake survivors with PTSD suggesting a greater severity among men. Interestingly, post-traumatic stress symptomatology appears to be a better correlate of these behaviors among women than among men, suggesting the need for further studies based on a gender approach.

## Introduction

Earthquakes are one of the most frequent natural disasters occurring across the world and, among exposed individuals, Post-Traumatic Stress Disorder (PTSD) represents the most frequently occurring psychiatric sequelae (Maj et al., 1989; Armenian et al., 2002; Lai et al., 2004; Jahangiri et al., 2010; Dell’Osso et al., 2011a; Hussain et al., 2011; Zhang et al., 2011; Ali et al., 2012).

There is increasing evidence in the literature on the specific clinical and neurobiological correlates of PTSD and particularly, on the specific gender differences in PTSD prevalence rates, with women being more affected than men across different cultures and at different ages (Armenian et al., 2000; Kessler, 2000; Green, 2003; Cohen, 2008; Liberzon and Sripada, 2008; Alhasnawi et al., 2009; Dell’Osso et al., 2010, 2011a,b; Pratchett et al., 2010; Friedman et al., 2011; Rabinak et al., 2011; Sherin and Nemeroff, 2011; Xu and Song, 2011). Increasing data agree on the tendency to a chronic course, higher incidence of alcohol and substance abuse and heightened risk for suicide in PTSD patients (Kessler, 2000; Krysinska and Lester, 2010; McFarlane, 2010; Jessup et al., 2012; Pietrzak et al., 2012). However, data on mass trauma survivors, particularly on earthquake victims, are scarce (Shimizu et al., 2000; Vlahov et al., 2002; Chang et al., 2005; Vehid et al., 2006; Cerdá et al., 2011; Dell’Osso et al., 2011a; Pollice et al., 2011; Stratta et al., 2012) and little is known on other possible maladaptive behaviors that may be developed and on any eventual gender differences (McMillen et al., 2000; Bal and Jensen, 2007; Goenjian et al., 2009; Cairo et al., 2010; Dell’Osso et al., 2011a, 2012).

Maladaptive behaviors are defined as volitional behaviors whose outcome is uncertain and which entail negative consequences that impact everyday activities (Irwin, 1990; Pat-Horenczyk et al., 2007; Hartley et al., 2008; Dell’Osso et al., 2012). Anecdotal data have been reported on maladaptive behaviors, such as risk-taking behaviors, dangerous driving, or promiscuous sex emerging in patients with PTSD. Aggressive and unsafe driving has been reported amongst male veterans of the Iraq and Afghanistan wars, with PTSD severity being associated to aggressive driving but not other forms of unsafe driving (Kuhn et al., 2010). Iraq and Afghanistan war veterans with PTSD also showed significantly higher rates of aggressive traits, including violent behaviors, compared to those without PTSD (Jakupcak et al., 2007; Elbogen et al., 2010). More data have been reported on risk-taking behaviors, such as smoking, alcohol, and substance use, car racing, weapon carrying, violence, and delinquency among adolescents and young adults with PTSD due to terrorism, fire, or violence. Interestingly, in these studies higher rates have been reported in victims with PTSD compared to non-affected subjects, with boys reporting significantly higher rates than girls (Glodich and Allen, 1998; Sugar, 1999; Gore-Feltun and Koopman, 2002; Steven et al., 2003). Considering the relevance of these conditions, particularly that of substance use disorders when co-occurring with mood or anxiety disorders (Sbrana et al., 2005; Bizzarri et al., 2007; Maremmani et al., 2012), further studies are warranted on PTSD.

Despite the fact that literature has been increasingly focused on post-traumatic stress reactions emerging in general population samples of earthquake adult survivors, to the best of our knowledge no study has yet explored gender differences in maladaptive behaviors occurring in these patients. On April 6th 2009, at 3:32 a.m., an earthquake of magnitude 6.3 on the Richter scale struck L’Aquila, Italy, a town with a population of 72,000 inhabitants, with a massive destroying effect on buildings, inducing the death of 309 people and the injury of more than 1,600. Furthermore, after this event continuous tremors have been going on in the town of L’Aquila and still present today. In a previous study on 512 students who survived the earthquake 10 months earlier (Dell’Osso et al., 2011a), we reported PTSD prevalence rates as high as 37.5%, with significantly higher rates of maladaptive behaviors among boys compared to girls.

Aims of the present study were to investigate the relationships between maladaptive behaviors and PTSD in earthquake survivors, besides the gender differences in the type and prevalence of maladaptive behaviors and their association with PTSD. With these aims in view, we investigated 900 L’Aquila residents who experienced the earthquake of April 6th 2009.

## Materials and Methods

### Study participants

The target population included residents of the town of L’Aquila, who lived in the urban area of the town and experienced the earthquake of April 6th 2009. Ten months earlier, all residents of the town of L’Aquila were directly exposed to the disaster, had received assistance in the emergency conditions that prevailed and were displaced in locations within a 150 km area from the town or in tents located in the urban area. Even 10 months after the earthquake, only 25% of the inhabitants were able to return to their homes.

The instruments were administered to an original sample of 939 subjects but complete data were available for 900 subjects, 446 women and 454 men. Within the whole sample, 372 survivors reported PTSD, 122 men and 250 women. Patients with PTSD had a mean age of 24.90 ± 12.15, 26.17 ± 13.6, 24.28 ± 11.35 years in the total sample and among men and women respectively.

Symptoms of post-traumatic stress related to the earthquake were explored on the Trauma and Loss Spectrum-Self Report (TALS-SR, Dell’Osso et al., 2008, 2009).

The Ethics Committee of the University of L’Aquila approved all recruitment and assessment procedures. Eligible subjects provided written informed consent after receiving a complete description of the study and having an opportunity to ask questions.

### Instruments and assessments

The TALS-SR was developed by researchers who comprise the Italian-American team belonging to the so-called *Spectrum-Project* (Frank et al., 1998; Cassano et al., 1999). The TALS-SR includes 116 items exploring the lifetime experience of a range of loss and/or traumatic events and lifetime symptoms, behaviors and personal characteristics that might represent manifestations and/or risk factors for the development of a stress response syndrome. Items responses are coded dichotomously (yes/no). The instrument is organized into nine domains and domain scores are obtained by counting the number of positive answers. In accordance with the aims of the present study subjects were asked to fulfill domains V, VI, VII, and VIII, referring symptoms that occurred after earthquake exposure. Domain V (“Re-experiencing”), domain VI (“Avoidance and numbing”), and domain VIII (“Arousal”) include re-experiencing, avoidance and numbing, and arousal symptoms respectively. Domain VII (“Maladaptive copying”) targets maladaptive coping and behaviors including: no self-care, scarce adherence to on-going medications, alcohol or drug abuse, risk-taking behaviors (dangerous driving, promiscuous sex, etc.), thoughts of death, suicidal ideations, and attempts. Each items explores whether these occurred after exposure to loss or trauma.

The presence of PTSD was assessed by means of the TALS-SR items corresponding to DSM-IV-TR criteria for PTSD diagnosis (Dell’Osso et al., 2011a,b). A diagnosis of partial PTSD was assessed when criteria B and or C or D for DSM-IV were satisfied. According to the aim of the present study we also analyzed the prevalence rates of endorsement of the items of the TALS-SR on domain VII.

In order to explore the association between maladaptive behaviors, gender, and PTSD, we adopted also a dichotomic variable indicating the presence of at least one of the maladaptive behaviors in each subject.

### Statistical analyses

We utilized the Chi-Square test to examine PTSD and gender differences in the rates of endorsement of maladaptive behaviors. Multiple logistic regression analyses were performed to: (1) study the association between gender and PTSD and their possible interaction in predicting the presence of at least one maladaptive behavior; (2) identify the maladaptive behaviors items that best predicted gender.

Pearson correlation coefficients were adopted to explore the association between PTSD symptoms, determined by means of the TALS-SR domains IV, V, and VI total scores, and maladaptive behaviors, determined by means of the TALS-SR domain VII total score.

All statistical analyses were carried out using Statistical Package for Social Science, version 16.0 (SPSS Inc., Chicago 2010).

## Results

Earthquake survivors with PTSD reported significantly higher prevalence rates of maladaptive behaviors than those without PTSD (see Table 1).

**Table 1:** **Maladaptive behaviors (TALS-SR Domain VII) prevalence rates in 900 L’Aquila earthquake survivors: PTSD vs. No PTSD**.

TALS-SR domain VII (maladaptive copying)	PTSD (*N* = 372) *N* (%)	No PTSD (*N* = 528) *N* (%)	χ^2^	*p*
97. …Stop taking care of yourself, for example, not getting enough rest or not eating right?	164 (44.1)	59 (11.2)	125.07	<0.001
98. …Stop taking prescribed medications or fail to follow-up with medical recommendations, such as appointments, diagnostic tests, or a diet?	39 (10.5)	14 (2.7)	22.77	<0.001
99. …Use alcohol or drugs or over-the-counter medications to calm yourself or to relieve emotional or physical pain?	87 (23.4)	59 (11.2)	22.94	<0.001
100. …Engage in risk-taking behaviors, such as driving fast, promiscuous sex, hanging out in dangerous neighborhoods?	55 (14.8)	44 (8.3)	8.63	=0.003
101. …Wish you hadn’t survived?	64 (17.2)	34 (6.4)	24.97	<0.001
102. …Think about ending your life?	32 (8.6)	15 (2.8)	13.57	<0.001
103. …Intentionally scratch, cut, burn, or hurt yourself?	34 (9.1)	15 (2.8)	15.62	<0.001
104. …Attempt suicide?	19 (5.1)	10 (1.9)	6.27	=0.012

A multiple logistic regression showed a statistically significant interaction gender * PTSD in predicting the presence of at least one maladaptive behavior (see Table 2). Indeed, the prevalence of at least one of the maladaptive behaviors is significantly higher in men than in women among survivors with PTSD only (69.4% vs. 56.6%, *p* = 0.024).

**Table 2:** **Binary logistic regression analysis (PTSD and gender as predictors of at least one maladaptive behavior) applied to 372 L’Aquila earthquake survivors with PTSD**.

	*B* (SE)	Odds ratio	C.I._95%_	*p*
Constant	−0.98 (0.13)			<0.001
PTSD	1.80 (0.23)	6.06	3.84–9.58	<0.001
Gender	0.13 (0.20)	1.14	0.77–1.68	=0.509
PTSD by Gender	−0.68 (0.31)	0.51	0.28–0.92	=0.026

(*χ^2^ = 101.60, p < 0.001, Cox R^2^ = 0.11, Negelkerke R^2^ = 0.14*).

*O.R., odds ratio; C.I., coefficient interval*.

In particular, men reported statistically significant higher endorsement rates in items *N =* 98 (“*Stop taking prescribed medications or fail to follow-up with medical recommendations…?*,” χ^2^ = 7.73; *p* = 0.005), *N* = 99 (“*Use alcohol or drugs or over-the-counter medications to calm yourself* …*?*,” χ^2^ = 8.19; *p* = 0.004), *N =* 100 (“*Engage in risk-taking behaviors…?*,” χ^2^ = 29.52; *p* < 0.001), *N =* 103 (“*Intentionally scratch, cut, burn or hurt yourself…?*,” χ^2^ = 12.84; *p* < 0.001), and *N =* 104 (“*Attempt suicide*…?,” χ^2^ = 26.80; *p* < 0.001) of the TALS-SR (see Table 3).

**Table 3:** **Maladaptive behaviors (TALS-SR Domain VII) prevalence rates in 372 L’Aquila earthquake survivors with PTSD: gender differences**.

TALS-SR Domain VII (Maladaptive copying)	Men (*N* = 122) *N* (%)	Women (*N* = 250) *N* (%)	χ^2^	*p*
97. …Stop taking care of yourself, for example, not getting enough rest or not eating right?	51 (41.8)	113 (45.2)	0.26	0.611
98. …Stop taking prescribed medications or fail to follow-up with medical recommendations, such as appointments, diagnostic tests, or a diet?	21 (17.2)	18 (7.2)	7.73	0.005
99. …Use alcohol or drugs or over-the-counter medications to calm yourself or to relieve emotional or physical pain?	40 (32.8)	47 (18.8)	8.19	0.004
100. …Engage in risk-taking behaviors, such as driving fast, promiscuous sex, hanging out in dangerous neighborhoods?	36 (29.5)	19 (7.6)	29.52	<0.001
101. …Wish you hadn’t survived?	23 (18.9)	41 (16.4)	0.20	0.658
102. …Think about ending your life?	15 (12.3)	17 (6.8)	2.451	0.117
103. …Intentionally scratch, cut, burn, or hurt yourself?	21 (17.2)	13 (5.2)	12.84	<0.001
104. …Attempt suicide?	17 (14.0)	2 (0.8)	26.80	<0.001

**Figure 1:**
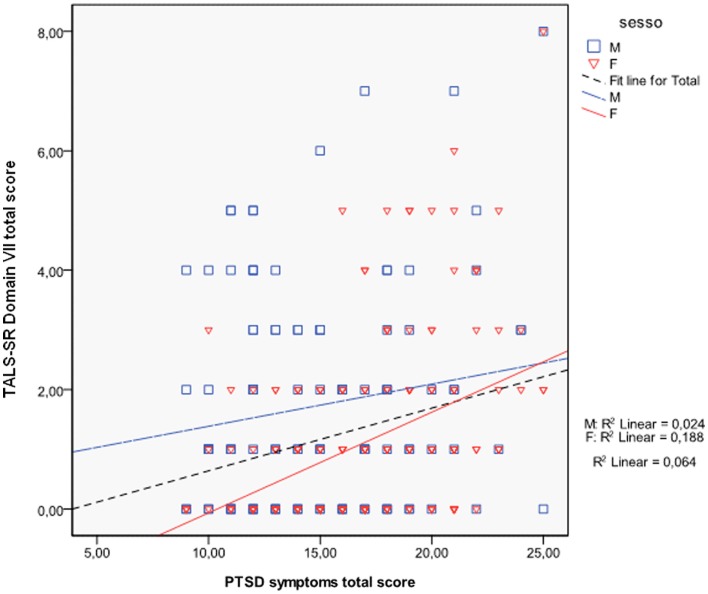
**Gender differences in the correlations between post-traumatic stress symptoms and TALS-SR domain VII total score**.

The Scattergram reported in Figure 1 shows the differential association between maladaptive behaviors, evaluated by adopting TALS-SR domain VII total scores, gender, and PTSD symptoms, evaluated by the sum of TALS-SR domains V (Re-experiencing), VI (Avoidance and numbing), and VIII scores (Arousal). In particular, a moderate Pearson correlation coefficient with PTSD symptoms, between moderate and good, emerged in women (*r* = 0.43, *p* < 0.001) but not in men (*r* = 0.15, *p* = 0.097). To note, in women significant correlations also emerged between maladaptive coping and each of the total scores of the three symptomatological domains (domain V Re-experiencing, *r* = 0.23, *p* < 0.001; domain VI Avoidance and numbing, *r* = 0.44, *p* < 0.001; domain VIII Arousal, *r* = 0.25, *p* < 0.001) while in men, a significant correlation emerged with domain VI only (*r* = 0.31 *p* = 0.001).

## Discussion

To the best of our knowledge, the present study is the first population-based study exploring gender impact on the maladaptive behavioral sequelae of a major earthquake. Unlike other studies that have explored maladaptive behaviors in special populations, such as veterans or adolescents (Glodich and Allen, 1998; Sugar, 1999; Gore-Feltun and Koopman, 2002; Steven et al., 2003; Jakupcak et al., 2007; Pat-Horenczyk et al., 2007; Elbogen et al., 2010; Kuhn et al., 2010; Dell’Osso et al., 2011a), we examined a sample of Italian civilian survivors to the earthquake that massively affected the town of L’Aquila in April 2009.

Our results indicate an alarming rate of self-reported maladaptive behaviors by L’Aquila earthquake survivors 10 months after the event occurred, with significantly higher rates among victims reporting PTSD compared to those without. In an exploration of substance abuse among young people exposed to this same event, Pollice et al. (2011) brought to light a marked increase in levels of abuse compared to prior to the trauma. Our data show significantly higher rates of alcohol or drug abuse in earthquake survivors with PTSD, reported in the aftermath of the event, with rates as high as more than 20%, compared to survivors who did not report PTSD presenting rates around 10%. Almost half of PTSD survivors also reported a decrease in self-care (e.g., not getting enough rest, not eating properly), with more than 10% reporting suspension of on-going treatments or medical recommendations. In line with previous studies on war veterans and adolescents (Glodich and Allen, 1998; Sugar, 1999; Gore-Feltun and Koopman, 2002; Steven et al., 2003; Jakupcak et al., 2007; Cepeda et al., 2010; Elbogen et al., 2010) we found significantly higher rates of risk-taking behaviors (such as unsafe driving, promiscuous sex) among survivors with PTSD. Further, more than one third of these subjects also reported at least one endorsement among items exploring suicidal ideation or attempts.

Significant gender differences emerged among survivors with PTSD, with men reporting significantly higher rates of maladaptive behaviors than women. In particular, we found that men reported significantly more than women interruption of on-going treatments, alcohol, or drug abuse, or over-the-counter medication use to calm themselves, risk-taking behaviors, including self-injuring and suicide attempts. In a previous study, some of our group (Rossi et al., 2011) compared the rates of new psychopharmacological prescriptions in the 6 months after the L’Aquila 2009 earthquake to the same period 1 year before, showing a 37% increase of new prescriptions for antidepressants and a 129% increase for antipsychotic prescriptions, with older age and female gender associated with the increased number of prescriptions.

Our results also suggest a significant association between some specific maladaptive behaviors, such as engagement in risk-taking behaviors and suicide attempts, and male gender in PTSD survivors. In this regard, in a sample of 409 Israeli adolescents exposed to recurrent terrorism, Pat-Horenczyk et al. (2007) reported significantly higher maladaptive behaviors, as a manifestation of functional impairment, in victims with PTSD, particularly among boys. Virtually all studies of the psychological effects of trauma indicate that women are more inclined to report anxiety and mood symptoms (Green, 2003; Shaw, 2003; Lai et al., 2004; Bal and Jensen, 2007; Cohen, 2008; Goenjian et al., 2009; Pratchett et al., 2010; Xu and Liao, 2011; Xu and Song, 2011; Zhang et al., 2011; Ali et al., 2012) and are at higher risk for developing post-traumatic distress than men (Davis and Siegel, 2000). However our data suggest that men tend to manifest more disruption in their behavioral adaption. Some authors in fact interpreted similar results in adolescents, suggesting that men tend to express psychological disturbances through acting out and external behavior, whereas women tend to express their distress by turning their feelings inwards, leading to depression and anxiety (Ostrov et al., 1989; Hirschberger et al., 2002; Pat-Horenczyk et al., 2007; Xu and He, 2012).

When looking at the possible associations between post-traumatic stress symptomatology and maladaptive behaviors significant correlations emerged in women, with each of the TALS-SR symptomatological domains, while the same was confirmed only for avoidance and numbing symptoms in men. Pat-Horenczyk et al. (2007) reported post-traumatic stress symptoms, including fear, re-experiencing, avoidance, and functional impairment, to be significant predictors of the severity of risk-taking behaviors beyond gender and level of exposure. Our results do not replicate these data as a significant association with post-traumatic stress symptoms emerged in women while in men this was confirmed for avoidance and numbing only.

Some important limitations of the study should be kept in mind before interpreting the results: first the limited number of subjects; second, the use of self-report instruments, instead of the rating of the clinician, in order to detect PTSD symptoms and even diagnosis. A self-report of PTSD symptoms may be, in fact, considered less accurate. The third limitation is the lack of information on the presence of Axis I psychiatric comorbidities and this may account for the answers on domain VII question regarding alcohol or drug abuse. Fourthly, in the present investigation we did not assess social support, though such support is generally believed to be high in Italy due to the closely knit family structure, and gender differences may also occur in this regard.

Despite these limitations, our results confirm the pervasive effects of a disaster, such as an earthquake, not only for mental health in the general population exposed, but also for the possible development of risk-taking behaviors that may affect men and women differently. In this regard, our results highlight the need to investigate such behaviors among earthquake exposed populations more in detail, with particular attention to gender differences, in order to get a more accurate understanding of post-traumatic stress psychopathology. Previous research (Armenian et al., 2000, 2002) has shown depression to be one of the major complications of earthquakes, possibly as a result of the multiple losses sustained. Given that women are vulnerable not only to PTSD, but also to anxiety disorders and major depression that can interfere and delay the recovery of the former, therapeutic intervention should be broadened beyond PTSD. Finally, the difference in maladaptive behaviors in men and women might be a correlate of the predominance of anxious-depressive symptomatology in women. In other words anxious depression might serve as a “break” on maladaptive behavior in women. These hypotheses deserve investigation in future analyses and studies.

## Conflict of Interest Statement

The authors declare that the research was conducted in the absence of any commercial or financial relationships that could be construed as a potential conflict of interest.
